# Artificial intelligence support in MR imaging of incidental renal masses: an early health technology assessment

**DOI:** 10.1007/s00330-024-10643-5

**Published:** 2024-02-23

**Authors:** Alexander W. Marka, Johanna Luitjens, Florian T. Gassert, Lisa Steinhelfer, Egon Burian, Johannes Rübenthaler, Vincent Schwarze, Matthias F. Froelich, Marcus R. Makowski, Felix G. Gassert

**Affiliations:** 1 Department of Diagnostic and Interventional Radiology, Klinikum Rechts Der Isar, School of Medicine, Technical University of Munich, Institut für diagnostische und interventionelle Radiologie, Ismaninger Str. 22, 81675 Munich, Germany; 2 Department of Radiology, Klinikum Großhadern, Ludwig-Maximilians-Universität, Marchioninistraße 15, 81377 Munich, Germany; 3 Department of Radiology and Nuclear Medicine, University Medical Center Mannheim, Heidelberg University, Theodor-Kutzer-Ufer 1-3, 68167 Mannheim, Germany

**Keywords:** Kidney, Incidental findings, Cost-effectiveness analysis, Artificial intelligence, MRI

## Abstract

**Objective:**

This study analyzes the potential cost-effectiveness of integrating an artificial intelligence (AI)–assisted system into the differentiation of incidental renal lesions as benign or malignant on MR images during follow-up.

**Materials and methods:**

For estimation of quality-adjusted life years (QALYs) and lifetime costs, a decision model was created, including the MRI strategy and MRI + AI strategy. Model input parameters were derived from recent literature. Willingness to pay (WTP) was set to $100,000/QALY. Costs of $0 for the AI were assumed in the base-case scenario. Model uncertainty and costs of the AI system were assessed using deterministic and probabilistic sensitivity analysis.

**Results:**

Average total costs were at $8054 for the MRI strategy and $7939 for additional use of an AI-based algorithm. The model yielded a cumulative effectiveness of 8.76 QALYs for the MRI strategy and of 8.77 for the MRI + AI strategy. The economically dominant strategy was MRI + AI. Deterministic and probabilistic sensitivity analysis showed high robustness of the model with the incremental cost-effectiveness ratio (ICER), which represents the incremental cost associated with one additional QALY gained, remaining below the WTP for variation of the input parameters. If increasing costs for the algorithm, the ICER of $0/QALY was exceeded at $115, and the defined WTP was exceeded at $667 for the use of the AI.

**Conclusions:**

This analysis, rooted in assumptions, suggests that the additional use of an AI-based algorithm may be a potentially cost-effective alternative in the differentiation of incidental renal lesions using MRI and needs to be confirmed in the future.

**Clinical relevance statement:**

These results hint at AI’s the potential impact on diagnosing renal masses. While the current study urges careful interpretation, ongoing research is essential to confirm and seamlessly integrate AI into clinical practice, ensuring its efficacy in routine diagnostics.

**Key Points:**

• *This is a model-based study using data from literature where AI has been applied in the diagnostic workup of incidental renal lesions.*

• *MRI + AI has the potential to be a cost-effective alternative in the differentiation of incidental renal lesions.*

• *The additional use of AI can reduce costs in the diagnostic workup of incidental renal lesions.*

## Introduction

The number of computed tomography (CT) and ultrasound examinations has nearly doubled within the last 10 years [1]. In about 13–27% of individuals undergoing abdominal imaging for other purposes, incidental renal lesions are found, and it is thought that more than 50% of patients over the age of 50 have a renal lesion [2]. With an increasing number of imaging procedures expected in the future and thus a higher number of incidental renal lesions, accurate and cost-effective differentiation between benign and malignant lesions is crucial.

Diagnostic pathways after detection of incidental renal lesions depend on the presence of cystic and solid components. The malignancy of cystic renal masses can be graded using the Bosniak Classification after contrast-enhanced magnetic resonance imaging (MRI) or CT [3]. Solid renal masses are most commonly further characterized using contrast-enhanced MRI, but ultrasound has also been proposed as accurate diagnostic means [4]. For histopathological proof of malignancy, suspicious lesions seen in imaging are either biopsied or directly surgically removed. Total nephrectomy can be considered for malignant solid renal masses bigger than 7 cm, and organ-preserving partial nephrectomy can be performed for minor lesions [5]. Follow-up is recommended, due to the risk of local recurrence, especially in the first year after treatment [6].

Convolutional neural networks (CNNs) have shown positive results in detection and differentiation of benign and malignant tumors, and are on par or even outperform sub-specialized experts in diagnostic performance [7, 8]. Xi et al developed a CNN and showed that it can effectively distinguish between benign and malignant renal lesions when using routine MR imaging with high accuracy, sensitivity, and specificity [8].

Although artificial intelligence (AI) has yielded promising outcomes, the implementation in clinical routine remains challenging. High investment costs are necessary for installation of necessary hardware and software [9]. The demonstration of both patient benefit and economic advantages could accelerate the translation into routine clinical practice. Ziegelmayer et al and van Leeuwen et al conducted cost-effectiveness analyses on AI support for lung cancer screening and early vessel occlusion detection, respectively [10, 11]. In addition to this, two literature reviews on health economic evaluations (HEEs) for AI-based health interventions underscore the increasing acceptance of AI in healthcare [12, 13]. Both reviews highlight the potential for enhanced outcomes and signify the escalating significance of AI in the field of radiology. So far, no study has been conducted comparing the use of neural networks in identifying malignant renal lesions to stand-alone MR imaging from an economic point of view. The objective of our analysis was to explore the possible cost-effectiveness of an AI-based system in distinguishing incidentally found renal lesions as benign or malignant. This study, while not a definitive technology assessment, provides insights into approaching these considerations based on a single publication. Additionally, it aims to define a cost margin for potential clinical integration from the healthcare sector perspective.

## Materials and methods

### Model structure

**Fig. 1:**
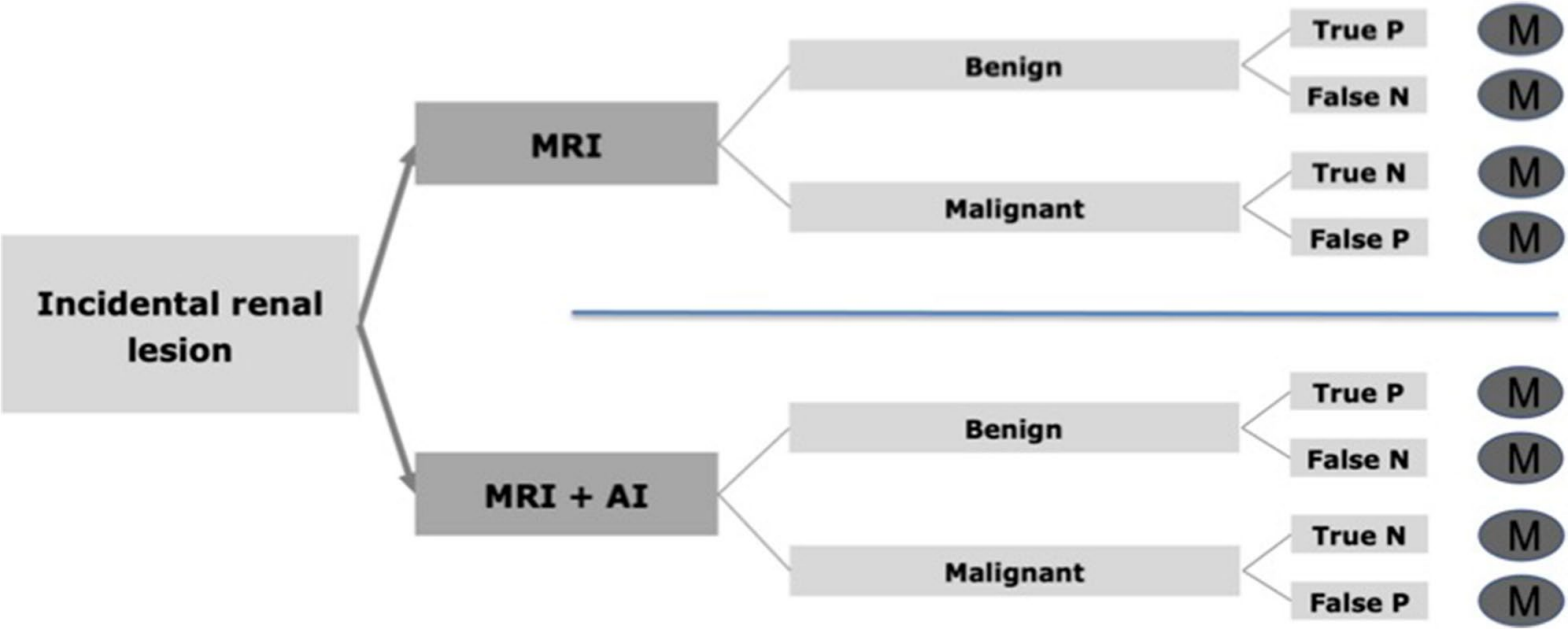
Economic model for the diagnostic options of the MRI strategy and the MRI + AI strategy. For each outcome a Markov model analysis was performed. AI, artificial intelligence; M, Markov model; MRI, magnetic resonance imaging; N, negative; P, positive

Based on the clinical scenario of an incidentally found renal mass, an economic model was created, including an MRI strategy and a MRI + AI strategy as diagnostic pathways, concluding in the different clinical scenarios (timely treatment, delayed treatment, no treatment, unnecessary diagnostics) which were simulated through a Markov model. The economic model is shown in Fig. 1.

The Markov model was designed using a specific decision-analytic software (TreeAge Pro Version 19.1.1, Williamstown, MA, USA). Based on the economic model and the respective accuracy of the diagnostic pathways, the starting point for the initial iteration was defined for every patient.

The Markov model was used for running iterations of possible disease outcomes. During each iterations, patients in each state are attributed certain costs and a certain quality of life (QoL) as well as transition probabilities to other states during the next iteration.

**Fig. 2:**
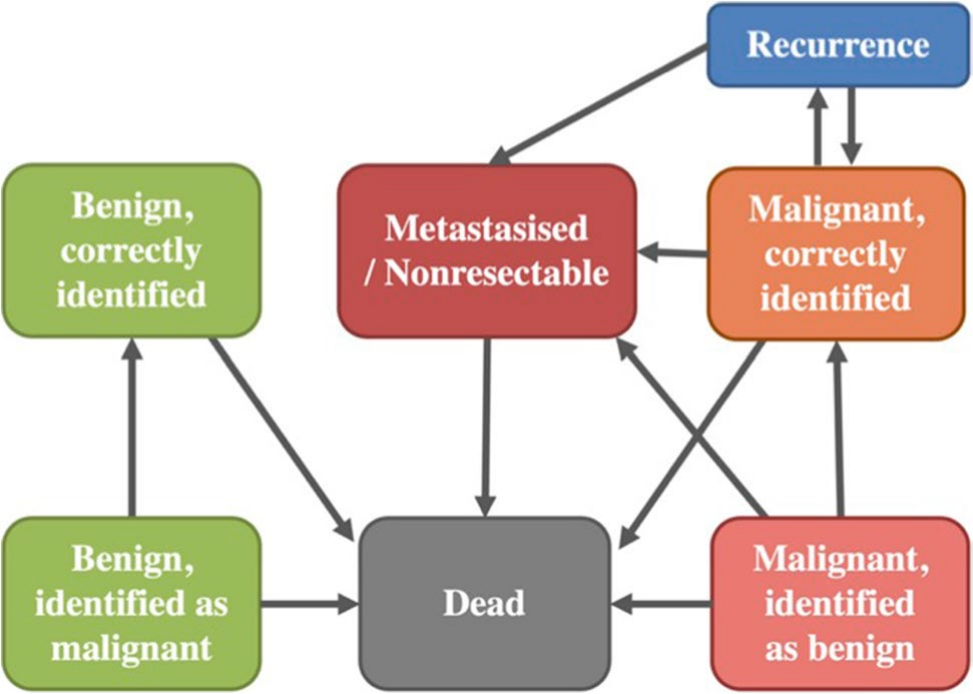
The Markov model with the respective states and their potential transition

The Markov model dedicated to renal carcinoma included the following states and is displayed in Fig. 2:

- Benign, correctly identified (patients without malignant renal mass = true negative)
- Benign, identified as malignant (patients without malignant renal mass, but suspicious = false positive)
- Malignant lesion, identified as benign (patients with undetected malignant renal mass = false negative)
- Malignant, correctly identified (patients with malignant renal mass and will be resected = true positive)
- Metastasized/non-resectable (patients with a malignant renal mass which is unresectable/palliative)
- Recurrence (Patients who show tumor recurrence after resection)
- Dead

The state “Recurrence” was created, as sometimes a malignant renal mass reoccurs after resection. During each iteration, a patient is in one of the abovementioned states. Between each iteration, the patient can transition to another state or remain within the same state with a certain probability. The possible transitions are shown as arrows between the states in Fig. 2. For example, a patient has a malignant renal lesion, which is detected as malignant (Malignant, correctly identified). This malignant renal lesion will be resected. In the next iteration, the patient can transition to the states “Recurrence,” “Metastasized/Non-resectable,” “Dead,” or not transition at all. According to Margulis et al, there is a probability of recurrence of 1.8% [14] and according to Manikandan et al a probability of occurrence of metastasis after resection of 0.18% [15] and an annual risk of death with a localized tumor according to the age-adjusted US lifetables [16]. These are examples of transition probabilities and further parameters are shown in Table 1.

**Table 1:** Input parameters

Variable		Estimate	Distribution	Source
Pre-test probability for malignancy		*0.19*	β	*O’Connor *et al [17]
Age at diagnostic procedure		*63 years*	β	Karakiewicz et al [18]
Assumed WTP		$100,000/QALY	β	Sanders et al [19]
Discount rate		3%	β	Sanders et al [19]
Markov model time		10 years	β	Assumption
**Diagnostic test performance**				
MR sensitivity		0.8	β	Xi et al [8]
MR specificity		0.35	β	Xi et al [8]
MR + AI sensitivity		0.92	β	Xi et al [8]
MR + AI specificity		0.41	β	Xi et al [8]
**Costs (acute)**				
Biopsy		$1535	γ	Medicare (Code: 50,200) [20]
MR		$453	γ	Medicare (Code: 74,182) [20]
ceCT		$246	γ	Medicare (Code: 74,170) [20]
Timely surgery + treatment (true positive)		$7652	γ	Medicare (Code: 52,355 and 50,543) [20]
No further action (true negative and false negative)		$0.00	γ	Assumption
Unnecessary surgery (false positive)		$4884	γ	Medicare (Code: 52,355) [20]
**Costs (long-term)**				
Yearly costs without tumor		$0	γ	Assumption
Yearly costs with localized (1st year)		$492	γ	Donat et al [21]
Yearly costs with localized (after 1st year)		$246	γ	Donat et al [21]
Yearly costs with metastatic (1st year)		$70,703	γ	Shih et al [22]
Yearly costs with metastatic (after 1st year)		$34,716	γ	Shih et al [22]
**Utilities**				
Alive, benign tumor		1	β	Assumption
After biopsy (= FP 1st year)		0.995	β	Adapted from Feldmann et al and Gassert et al [23, 24]
Alive, resected, 1st year (including resection)		0.97	β	Adapted from Jiang et al [25]
Alive, resected from 2nd year on		1	β	Adapted from Rossi et al [26]
Alive, non-resected		1	β	Assumption
Alive, resected, occurrence of metastasis		0.66	β	de Groot et al [27]
QoL after biopsy for 1 month		0.995	β	Adapted from Feldmann et al [23]
QoL after surgery for 1 month		0.7	β	Kim et al als Referenzpunkt (SF 12 Physical Component Score) [28]
Annual risk of death without malignancy		0	β	Assumption
**Transition probabilities**				
Probability of initial non-R0 resection		5.7%	β	Orosco et al [29]
Risk of metastases in false negative patients		1.0%	β	Bensalah et al [30]
Probability of local recurrence after resection		1.8%	β	Margulis et al [14]
Yearly probability of occurrence of metastases after resection		0.18%	β	Manikandan et al [15]
Probability of successful surgery of local recurrence		41.2%	β	Thomas et al [31]
Annual risk of death with localized tumor		Age adjusted	β	US Life Tables [16]
Death due to surgery		0%	β	Chaudery et al [32]
Detection of initially undetected localized lesion		100%	β	Assumption
Risk of death with metastasis		33.5%	β	Howlader et al [33]

*AI* artificial intelligence, *ceCT* contrast-enhanced computer tomography, *ICER* incremental cost-effectiveness ratio, *QALY* quality-adjusted life year, *QoL* quality of life, *WTP* willingness-to-pay

The Markov model runtime was set to 10 years with a duration per iteration of 1 year, resulting in a total of 10 iterations.

### Population

The population in this study is based on Xi et al [8]. In their study, patients with renal lesions confirmed by histology or imaging were retrospectively identified from two prominent academic centers in the United States (US) (HUP and MAY), two hospitals in the People’s Republic of China (SXH and PHH), and the Cancer Imaging Archive (TCIA) [8]. Most patients with renal lesions came from the academic centers in the US. Therefore, further data and literature included in this evaluation was based on the US healthcare system. Although this algorithm could be used in any kind of medical institution, our assumption will most likely hold true for academic centers, where further evaluations and handling of renal tumors is performed.

### Input parameters

Model input parameter included pre-test probability of a malignant lesion present, and mean age at the diagnostic procedure as well as diagnostic accuracy of the MRI strategy and the MRI + AI strategy. For the Markov model simulation, costs and utilities (measured as QoL) per iteration as well as transition probabilities between states were retrieved through review of current literature. These parameters are displayed in Table 1.

### Diagnostic test performances

Sensitivity and specificity for the correct classification of incidental renal lesions through a MRI strategy and a MRI + AI strategy are based on the study by Xi et al [8]. In this investigation, a multicenter cohort of 1162 renal lesions with a confirmed pathology or imaging diagnosis (655 of which were malignant and 507 of which were benign) were separated into training, validation, and test sets (70:20:10). A bagging classifier was used to build an ensemble model based on ResNet that included clinical factors with T1C and T2WI MR images to predict the pathology of renal tumors. Performance of the final model was evaluated against professional analysis. The ensemble deep learning model outperformed the average performance of the four experts in terms of sensitivity (0.92 vs. 0.80), and specificity (0.41 vs. 0.35) [8].

### Costs

Starting from a US healthcare perspective, costs were estimated based on Medicare data and available literature (Table 1). It was assumed that if a localized malignant tumor was diagnosed, surgery would follow in all cases. Costs of timely surgery and resection of a malignant renal tumor were set to USD 7652 (Medicare codes 52,355 and 50,543) [20]. After treatment, the long-term yearly costs of follow-up were estimated at USD 492 (corresponding to two contrast-enhanced CT scans) in the first year after resection and at USD 246 (corresponding to one contrast-enhanced CT scan) for every further year [21]. According to Shih et al, costs for patients with a metastasized renal tumor were at USD 70,703 for the first year after diagnosis and at USD 34,716 for every following year [22]. In patients with false positive results, only the costs of surgery were attributed with USD 4884.

### Utilities

For determination of the overall effectiveness, quality-adjusted life years (QALY) gained from each diagnostic procedure were calculated based on the QoL in each disease state. QoL was set to 1 for healthy patients as well as patients with an undetected malignant renal mass, as we assumed the patient to change disease state in the Markov model if the lesion became symptomatic. Due to the possible complications associated with surgery, QoL in the first year after resection of localized tumor was set to 0.97 [25]. However, according to previous literature, from the second year after resection QoL was set to 1 again [26]. In a metastasized disease state, QoL was set to 0.66 [27].

### Transition probabilities

Transition probabilities were derived from recent literature and are displayed in Table 1. The probability of detection of an initially undetected malignant renal mass was assumed to be at 100% after 1 year. The probability of occurrence of metastases in patients with a localized tumor was assumed to be 1%, which corresponds to the risk of metastases in false negatively diagnosed lesions [30]. The annual probability of occurrence of metastases after resection of a localized renal malignancy was set to 0.18%, based on recent literature [15]. As metastasis was thought to be the cause of the disease’s deadly outcome, the risk of death with a localized renal malignancy was assumed to be comparable to the risk of death without a malignant renal mass [33]. The age-dependent annual risk of death with localized renal malignancy was based on the current US Life Tables [16].

### Cost-effectiveness analysis

Cost-effectiveness analysis was performed using Markov simulations with a run time of 10 years after the detection of solid renal masses. The discount rate was set to 3.0% and willingness-to-pay (WTP) was set to USD 100,000 per QALY, as recommended in current guidelines. Willingness-to-pay is the valuation of health benefit in monetary terms and defined as the costs a society or healthcare system is willing to pay for an additional QALY gained [19]. In the base-case scenario, additional costs for the MRI + AI strategy as compared to the MRI strategy were set to USD 0 as this parameter was to be determined by the analysis. A deterministic sensitivity analysis of costs and diagnostic parameters was performed to evaluate model uncertainty and impact of variation of those parameters on the potential costs of the AI. These results were presented in a tornado diagram and given as a difference of incremental cost-effectiveness ratio (ICER) compared to the MRI strategy. The ICER is defined as the difference in cost between two possible interventions, divided by the difference in their effect and therefore represents the incremental cost associated with one additional QALY gained [19]. A probabilistic sensitivity analysis based on Monte Carlo simulations with 30,000 iterations was performed to show the individual patients outcomes based on the individual variation of the input parameters for evaluation of the robustness of the model. A threshold analysis was performed to define the maximum costs of the AI algorithm at different values for the WTP.

## Results

### Cost-effectiveness analysis

In the base-case scenario over a period of 10 years, total costs were at $8054 for the MRI strategy and at $7939 for the MRI + AI strategy if additional diagnostic costs for the use of AI were at USD 0. In the same scenario, the model yielded a cumulative effectiveness of 8.76 QALYs for the MRI strategy and a cumulative effectiveness of 8.77 for the MRI + AI strategy. Therefore, in the base-case scenario, the use of AI was the dominant strategy from a cost-effectiveness point of view.

### Probabilistic sensitivity analysis

**Fig. 3:**
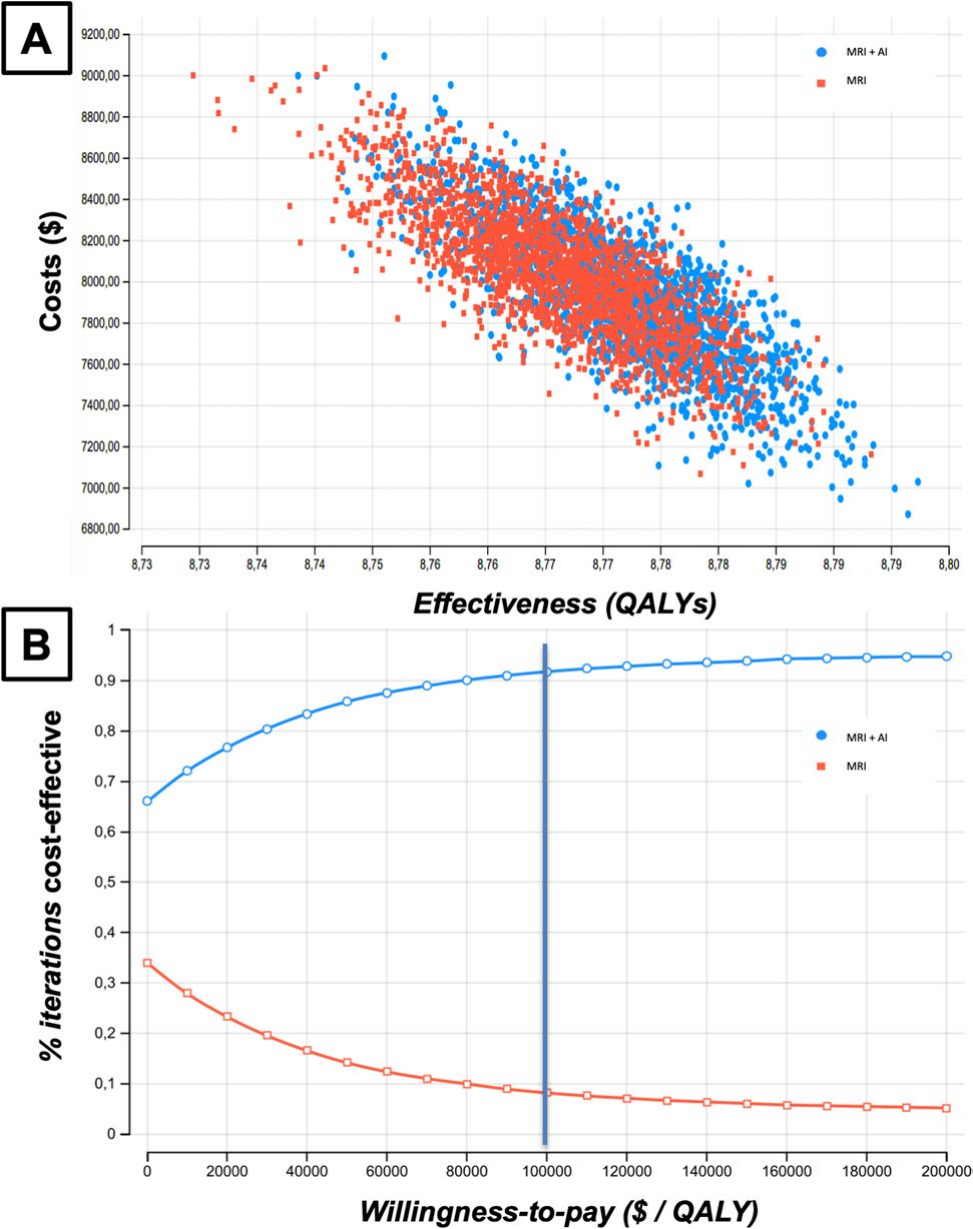
Scatterplot of Effectiveness and cost of the scenario “MRI” vs the scenario “MRI + AI” for 30,000 exemplary iterations (**A**). Although there is quite an overlap between the two scenarios, overall the iterations of the scenario “MRI + AI” show higher effectiveness and lower costs. Cost-effectiveness acceptability curve for a Willingness-to-pay threshold ranging from $0/QALY to $200,000/QALY (**B**). The base case scenario at $100,000/QALY is indicated by the blue bar. Results show that in the base case scenario, a majority of the iterations for the scenario “MRI + AI” are cost-effective. MRI, magnetic resonance imaging; AI, artificial intelligence; QALY, quality-adjusted life year; WTP, willingness-to-pay

The outcomes of the 30,000 iterations are shown in Fig. 3A, a scatterplot depicting the effectiveness and cost of the scenario “MRI” (red) versus the scenario “MRI + AI” (blue). Visualization reveals that, overall, iterations in the diagnostic pathway of the MRI strategy are more expensive and less effective compared to iterations in the diagnostic pathway of the MRI + AI strategy. Figure 3B shows the cost-effectiveness acceptability curve, which indicates the proportion of iterations, which are cost-effective for the MRI + AI strategy based on a variation of the WTP threshold. In the range of USD 0/QALY to USD 200,000/QALY, more than 50% of the iterations are cost-effective for the MRI + AI strategy as compared to MRI strategy.

### Deterministic sensitivity analysis

**Fig. 4:**
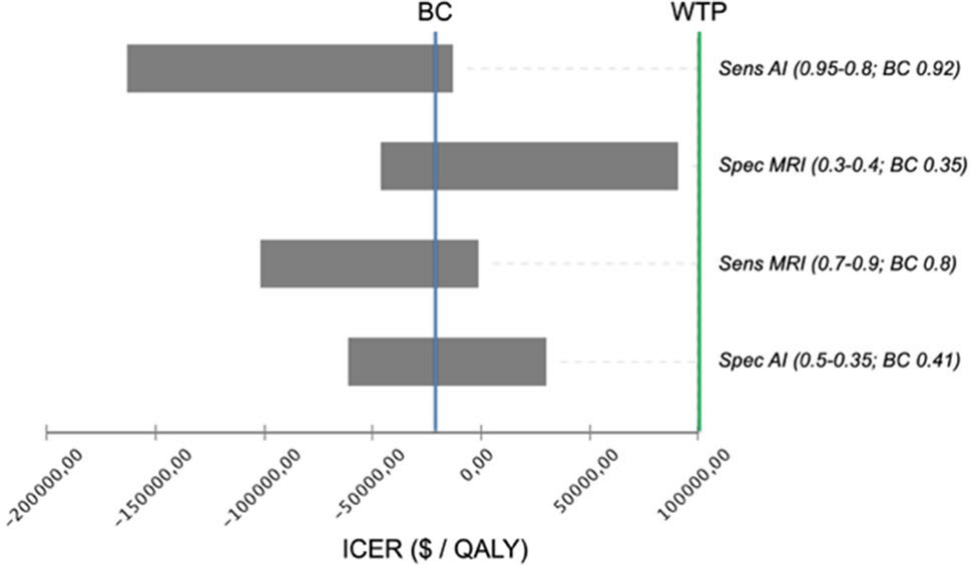
Deterministic sensitivity analysis presented as a tornado diagram (MRI + AI strategy vs. MRI strategy), showing how the variation of input parameters influences the incremental cost-effectiveness ratio in the base case scenario. The expected value in the base case scenario is marked with a blue line and the willingness-to-pay of $100,000/QALY with a green line. BC, base case; Sens, sensitivity; Spec, specificity; MRI, magnetic resonance imaging; AI, artificial intelligence; ICER, incremental cost-effectiveness ratio; QALY, quality-adjusted life year; WTP, willingness-to-pay

A deterministic sensitivity analysis was performed to account for variation of input parameters in literature. The results are shown as a tornado diagram in Fig. 4. ICER is positive if specificity of the MRI strategy increases above 0.38 and specificity of the MRI + AI strategy decreases below 0.39. However, within the indicated ranges of the input parameter, ICER stayed below the WTP threshold of USD 100,000/QALY in all cases.

### Threshold analysis

**Fig. 5:**
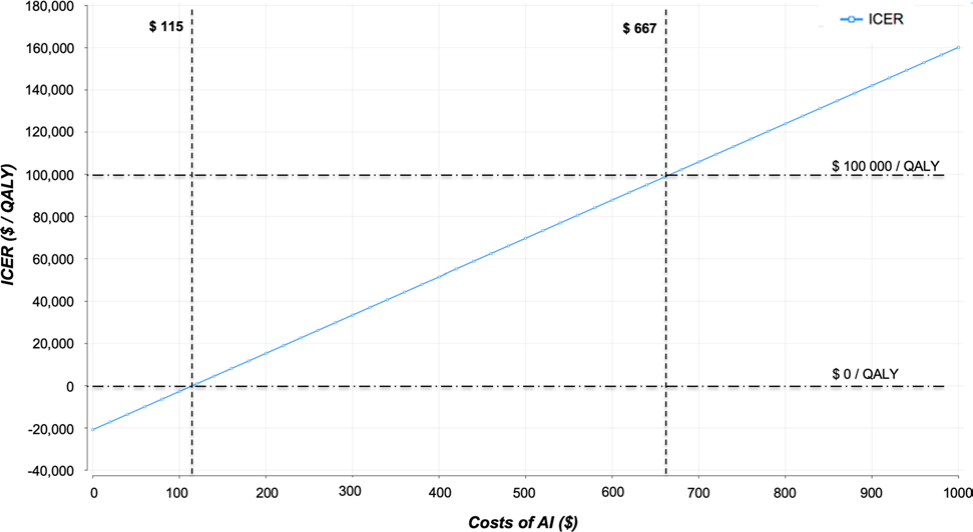
Threshold analysis showing maximum cost for the use of AI dependent on the underlying WTP threshold. AI, artificial intelligence; ICER, incremental cost-effectiveness ratio; QALY, quality-adjusted life year; WTP, willingness-to-pay

A threshold analysis was performed to define the maximum costs of the AI algorithm at different WTP thresholds. If additional costs for the use of AI as compared to the MRI strategy remained below USD 115 per application, the MRI + AI strategy remains the dominant strategy. When increasing the costs for the use of AI further, the assumed WTP of USD 100,000/QALY was only reached at USD 667 per application. Values for the maximum costs of AI at other WTP thresholds are depicted in Fig. 5.

## Discussion

This study emphasizes the potential cost-effectiveness rather than providing a definitive technology assessment. It indicates that integrating a deep learning–based diagnostic support (with a sensitivity of 0.92 and specificity of 0.41) into routine MR imaging is a cost-effective alternative compared to using MRI alone for distinguishing benign and malignant incidental renal lesions. Acknowledging the underlying assumptions, higher accuracies could potentially decrease the need for biopsies or surgical resections of benign renal lesions, ultimately lowering costs and improving patient outcomes by reducing the risk of delayed detection of malignant renal lesions. This is reciprocated by our study as the MRI + AI strategy remained the dominant strategy up to the threshold of $115. At a WTP of $100,000/QALY, additional cost of AI may be as high as $667 to remain the cost-effective alternative compared to the use of MRI alone.

Preoperative differentiation between benign and malignant renal lesions using non-invasive imaging techniques is a crucial factor for treatment planning but remains challenging from imaging. According to current clinical guidelines, suspicious renal lesions should be analyzed histopathologically via biopsy or resection [5]. Several studies can be found in literature using traditional machine-learning techniques, such as support vector machine and random forest, to distinguish incidental renal lesions based on CT radiomics [34, 35]. The CNN developed by Xi et al is one of the first studies using deep learning for differentiation of these lesions. If adapted into routine clinical practice, it has the potential to reduce the number of unnecessary diagnostics through more accurate classification [8]. However, to this date, there is not yet an AI product commercially available in Europe [36] or the United States [37] for this purpose.

To our knowledge, currently there are very few studies that tested the cost-effectiveness of AI-based algorithms in radiology. A study conducted by Ziegelmayer et al showed that the use of a 3D-convolutional neural network can be cost-effective when applied to CT-based lung cancer screening [11]. Similar to the current study, where the MRI + AI strategy remained the dominant strategy up to a threshold of $115 per patient, additional AI support in lung cancer screening remained the dominant strategy to a threshold of $68 [11]. Furthermore, van Leeuwen et al conducted a cost effectiveness-analysis on AI support for the early detection of vessel occlusion in the brain [10]. This study also showed that AI support is cost-effective and can reduce the number of missed large vessel occlusion with lower costs (− $156) and higher effectiveness (0.0095 QALYs). Van Leeuwen et al discuss the importance of using such CEAs to deduce the potential patient outcome and the benefit of an AI system to help not only its development, but also the translation into routine clinical practice [10]. In a related context, Vithlani et al and Jiao et al conducted comprehensive literature reviews, shedding light on numerous HEEs associated with AI-based health interventions [12, 13]. Their findings consistently revealed potential positive impacts on health outcomes. Notably, both manuscripts underscored the imperative for increased CEAs to effectively bridge the gap between the rapid progress of AI in healthcare and its practical application in real-world settings [12, 13]. Overall, these studies show that use of additional AI-based algorithms in routine imaging techniques can be beneficial in terms of both patient outcome and economic aspects when implemented into clinical diagnostics.

This study has limitations. Firstly, our model assumes the quality of life in a patient with a non-resected malignant tumor, as a study determining the quality of life in an unresected known malignant tumor would be unethical and therefore does not exist. Additionally, our focus is solely on MRI, excluding other techniques like contrast-enhanced ultrasound (CEUS) and contrast-enhanced CT, which have been proven to be a feasible diagnostic method in incidental renal lesions [38, 39]. However, the goal of this study was to evaluate the use of AI in diagnostic imaging, particular in MR imaging. To our knowledge, no algorithm has been developed so far to differentiate between benign and malignant renal lesions in imaging modalities other than MRI. Furthermore, the imaging quality and therefore diagnostic outcome of CEUS is dependent of the clinician’s skill set and the availability.

Moreover, while MRI is not commonly the initial choice for detecting incidental renal lesions, it is recommended, especially in cases of indeterminate renal masses, according to the ACR incidental findings committee [40]. It is important to note that no single imaging method can definitively distinguish between benign and malignant renal lesions. Nevertheless, the incorporation of AI alongside MRI has demonstrated encouraging outcomes, justifying our choice of this imaging approach. Furthermore, we have not incorporated alternative techniques aimed at enhancing the effectiveness of malignant detection in MRI, such as the multi-parametric MRI approach explored by Suresh de Silva et al [41]. This is because our primary emphasis in this paper is on AI, and currently, there is insufficient available data concerning novel methodologies.

Additionally, while deterministic sensitivity analysis may consider some parameter change, recommendations for each individual situation cannot be obtained from the model since cost-effectiveness analysis using decision-based models depends heavily on the input parameters. Furthermore, AI performance metrics in this study are based on a single study from Xi et al of a research algorithm tested on a split from the dataset it was trained on [8]. Published data on AI algorithms can vary widely depending on the study design, training cohort, and scanners. Also, the majority of the cohort in Xi et al was from the US with a few patientsfrom China. Despite Xi et al being the biggest study that compared AI performance with four expert radiologists in the differentiation of incidental renal lesions, future studies on this topic may show different results [8]. Several studies have assessed the effectiveness of AI in distinguishing between benign and malignant renal masses [42–44]. For instance, Maasa’a et al employed MRI-based radiomics and machine learning to assess the differentiation of 182 renal lesions in 160 patients [43]. Similarly, Wentland et al compared CT-based radiomics and machine learning with radiologist interpretation focusing on 148 renal masses [44]. Prioritizing realism, we chose Xi et al’s research for its large multicentric cohort in our investigation [8], opting for a specific example due to the challenges of integrating outcomes from multiple studies in this research context.

In conclusion, our study suggests that the potential integration of an AI-based algorithm could be a valuable tool for differentiating benign and malignant incidental renal lesions, considering patient benefits and economic factors. However, it is crucial to recognize that our analysis, rooted in various assumptions and diagnostic accuracy uncertainties, serves as an example for approaching these considerations in a hypothetical scenario based on a single publication. Establishing a benchmark for potential costs associated with AI application may contribute to the accelerated translation of such systems into clinical routine, bearing in mind the speculative nature of our findings.

## Abbreviations

AI: Artificial Intelligence
CEA: Cost-effectiveness analysis
CEUS: Contrast-enhanced ultrasound
CNN: Convolutional neural networks
CT: Computed tomography
HEE: Health economic evaluation
ICER: Incremental cost-effectiveness ratio
MRI: Magnetic resonance imaging
QALYs: Quality-adjusted life years
QoL: Quality of life
US: United States
WTP: Willingness to pay

## Declarations

### Guarantor

The scientific guarantor of this publication is Felix G. Gassert (Department of diagnostic and interventional Radiology, Klinikum rechts der Isar, School of Medicine, Technical University of Munich, Ismaninger Str. 22, 81675 Munich, Germany). Email: felix.gassert@tum.de.

### Statistics and biometry

One of the authors (FG) has significant statistical expertise.

### Methodology

• cost-effectiveness

• performed at one institution
